# Dual-Mode Reconfigurable Frequency-Selective Surface for Switching Between Narrowband and Wideband Applications

**DOI:** 10.3390/mi16091030

**Published:** 2025-09-08

**Authors:** Batuhan Uslu, Sena Esen Bayer Keskin, Nurhan Türker Tokan

**Affiliations:** 1 Department of Electrical and Electronics Engineering, Kırklareli University, Kırklareli 39100, Türkiye; batuhanuslu@klu.edu.tr; 2 Department of Electronics and Communication Engineering, Yildiz Technical University, Istanbul 34220, Türkiye; nturker@yildiz.edu.tr

**Keywords:** dual-mode, wideband, reconfigurable, FSS, PIN-diode, dynamic switching

## Abstract

This study presents a reconfigurable frequency-selective surface (R-FSS) designed to dynamically switch between WLAN, WiMAX, and sub-6 GHz band frequencies. The electronic switching mechanism of this R-FSS is controlled in real-time using PIN-diodes. Depending on the activation state of these diodes, the structure operates in three distinct modes. Among the three modes, one exhibits polarization-stable wideband suppression, whereas the other two demonstrate polarization selectivity by interchanging between the dual-narrow and single-wide stopband regimes under orthogonal polarizations. The design is described with an equivalent-circuit model, corroborated by full-wave electromagnetic simulations, and validated through measurements of a fabricated prototype. This reconfigurability allows the proposed structure to operate across WLAN, sub-6 GHz, and WiMAX frequency ranges either with two narrow stopbands or with a single-wide stopband, while providing polarization selectivity for frequency-selective applications.

## 1. Introduction

Frequency-selective surfaces (FSSs) are specifically designed to exhibit a frequency-dependent electromagnetic response, enabling them to selectively transmit desired electromagnetic signals while simultaneously attenuating or reflecting unwanted frequencies [1,2,3]. Today, FSSs are widely used for various purposes, including band-stop [4,5,6], band-pass [7,8,9], and absorptive functionalities [10,11,12,13], as well as for beam steering applications [14,15,16]. On the other hand, there is a growing demand for real-time frequency reconfigurability in FSSs. This is especially valid in critical scenarios, such as emergencies (e.g., earthquakes, disasters), or for enhancing data security. This need has driven the development of reconfigurable FSSs (R-FSSs), which are capable of dynamic frequency switching. R-FSSs achieve this reconfigurability by integrating active circuit elements such as Micro-Electromechanical Systems (MEMSs) [17,18], PIN-diodes [19,20,21,22], varactors [23,24], and liquid crystals [25,26]. PIN-diodes are preferred for their efficient switching, easy integration, and cost-effectiveness, making them ideal for reconfigurable frequency-selective surfaces. Their rapid switching speed and low insertion loss provide advantages over alternatives like varactors or MEMS, especially in broadband electronic switching and multiband shielding applications. In addition, PIN-diodes facilitate dynamic frequency control in frequency-selective rasorbers and RFID tag antennas by enabling state changes [27,28]. Additionally, rapid switching speed and low insertion loss of PIN-diodes make them suitable for broadband electronic switches and multiband shielding applications [29,30].

Modern wireless systems operate over an increasing number of frequency bands and applications, rendering conventional FSS designs insufficient. This limitation is particularly evident in Wireless Local Area Networks (WLANs), Worldwide Interoperability for Microwave Access (WiMAX), and sub-6 GHz applications [31], which inherently utilize multiple, distinct frequency bands. While such characteristics enable broader coverage, they also introduce spectrum congestion and inter-band interference. Consequently, there is an urgent need for filtering solutions capable of selectively suppressing unwanted electromagnetic waves within these critical frequency ranges. Furthermore, traditional FSS structures present design and fabrication challenges, including polarization-dependent responses in Transverse Electric/Transverse Magnetic (TE/TM) modes and complexities arising from increased number of layers or intricate geometries [32]. The proposed R-FSS addresses these limitations by offering the ability to switch between different frequency bands without requiring physical alteration. These adaptive features enable a much more efficient utilization of the existing spectrum, leading to time and cost savings in surface design processes. Current literature on R-FSSs predominantly focuses on designs offering reconfigurable band-stop [33,34], band-pass [35,36], absorptive [37,38], phase shifting and polarization-selective [39,40] or permeability characteristic switching [41,42,43]. A crucial void persists in designs that offer dynamic reconfigurability between distinct operational modes. Current research largely addresses multiband, wideband, or actively tunable (though often single or narrowband) FSS capabilities. However, the ability to actively switch between two narrow stopbands and a single-wide stopband, while simultaneously maintaining frequency tunability, remains an unfulfilled requirement.

In this study, a R-FSS design capable of actively switching from two narrow stopbands to a wide stopband with tunable frequency characteristics is presented. The proposed structure can effectively suppress the n79 band and part of the n96/n102 bands (narrow bands) in one mode, while in the other mode, it suppresses the n46, WiMAX, and WLAN bands (wideband). In this respect, the proposed design not only provides hardware simplification and cost advantages but also offers high flexibility for applications such as spectrum management and electromagnetic interference control. Especially for modern communication systems such as 5G/6G, WiMAX, and WLAN, this structure, which can rapidly adapt to different operating conditions, fills a gap in the literature and presents a solution for next-generation communication technologies.

## 2. Design and Analysis of the R-FSS

### 2.1. Proposed R-FSS Unit Cell Design

**Figure 1:**
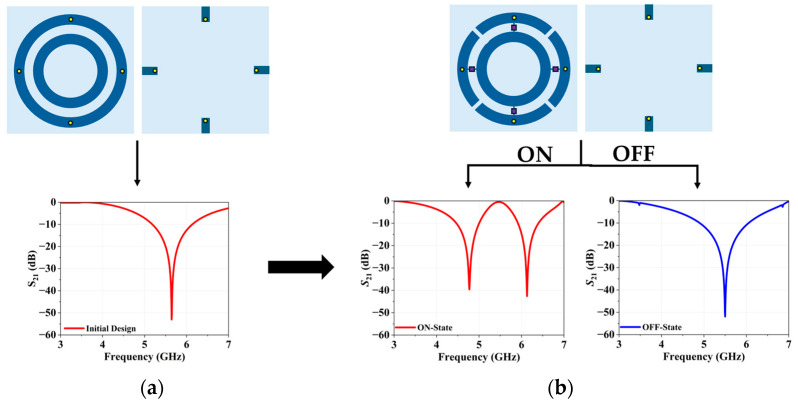
Design phases of the R-FSS: (**a**) initial design, (**b**) proposed design.

Figure 1 illustrates the unit cell of the R-FSS. The preliminary unit cell design given in Figure 1a consists of two closed circular ring elements, where the outer rings of the R-FSS are connected to each other through vias and connection lines. With this structure, the dual-band characteristic that attenuates the signals in the 1.81–2.19 GHz and 4.91–6.03 GHz frequency bands while allowing other microwave signals to transmit effectively is achieved. As reported in the literature, bringing these rings closer induces coupling effects, merging the dual resonances into a single, broader frequency band. As shown in Figure 1b, the electrical connection between the rings is dynamically controlled using PIN-diodes, enabling reconfigurable switching between narrowband and wideband frequency characteristics. To facilitate proper biasing of the PIN-diodes, rectangular patches are integrated on the backside of the substrate, connecting to the outer ring through vias. Each unit cell incorporates a total of four PIN-diodes, arranged in pairs oriented horizontally and vertically. Small notches are etched onto the outer ring to prevent short circuits during diode biasing, ensuring synchronized switching of diodes in either the ON or OFF states along the horizontal or vertical axes.

**Figure 2:**
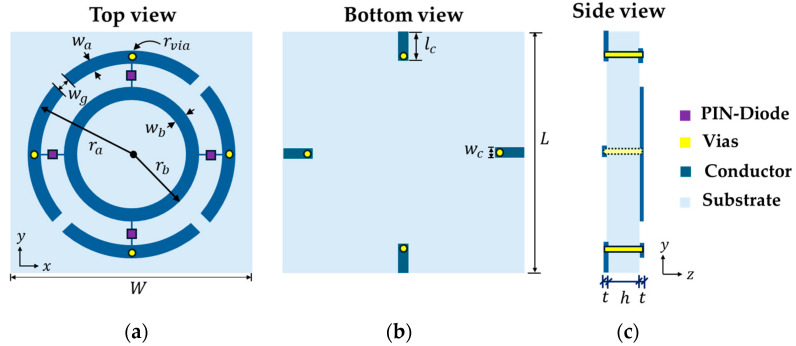
Geometry of the R-FSS: (**a**) top view, (**b**) bottom view, and (**c**) side view.

Figure 2 illustrates the design parameters of the proposed R-FSS, with the purple rectangles representing the PIN-diodes and the yellow circles indicating the vias. The electrical connection from the outer ring to the bottom layer is provided by vias with radius $r_{via}$, shown in yellow. The top layer consists of two concentric rings with radii $r_{a}$ and $r_{b}$, and corresponding patch widths $w_{a}$ and $w_{b}$.

**Figure 3:**
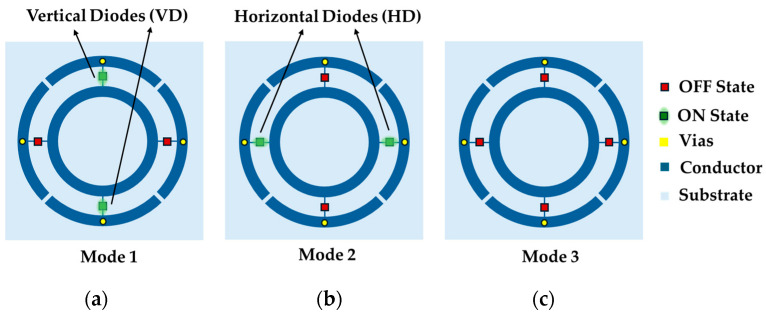
Modes and corresponding diode states of the R-FSS: (**a**) Mode 1, (**b**) Mode 2, and (**c**) Mode 3.

Each unit cell contains two horizontally and two vertically placed PIN-diodes. The horizontally placed diodes are labeled HD, while the vertically placed ones are labeled VD, as shown in Figure 3. In the figure, ON-state diodes are highlighted with green rectangles, whereas OFF-state diodes are shown in red. The proposed R-FSS has three operating modes: Mode 1, where the vertical diodes (VDs) are ON State; Mode 2, where the horizontal diodes (HDs) are ON State; and Mode 3, where all diodes are OFF state. The modes and corresponding states are summarized in Table 1.

As illustrated in Figure 3, two diodes are placed vertically and two horizontally between the concentric rings, ensuring that for any incident polarization, two diodes align with the TE field direction while the other two remain perpendicular to the TM field. Consequently, the proposed R-FSS demonstrates two distinct band-stop responses for TE and TM modes, corresponding to incident electric fields along the y- and x-directions, respectively. Due to the perpendicular orientation of the electric field relative to the PIN-diode leads, the switching effect is negligible. For TE polarization, the HDs do not influence the switching behavior, and the VDs remain the same in both Mode 2 and Mode 3, resulting in identical transmission characteristics. Similarly, for TM polarization, the VDs do not influence the switching operation, and the HDs remain the same in both Mode 1 and Mode 2, resulting in identical transmission characteristics. This inherent behavior of the PIN-diodes ensures polarization control across the modes.

### 2.2. Parametric Analyses

**Figure 4:**
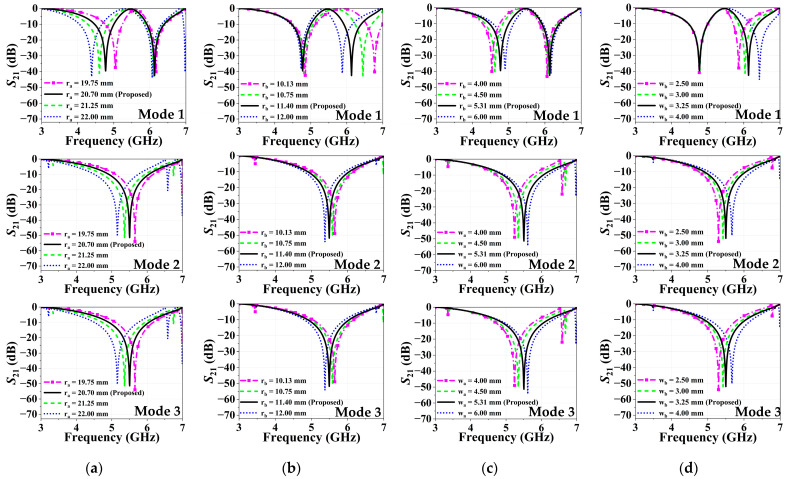
Transmission characteristics of the R-FSS in TE polarization: (**a**) $r_{a}$, (**b**) $r_{b}$, (**c**) $w_{a}$, and (**d**) $w_{b}$.

**Figure 5:**
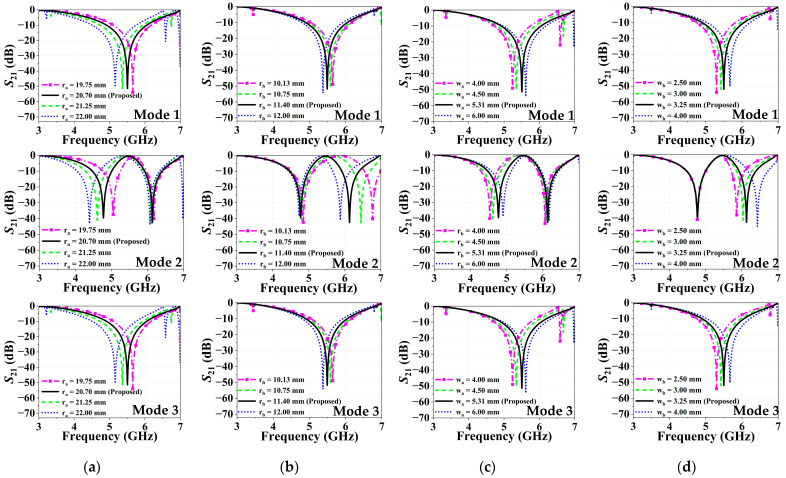
Transmission characteristics of the R-FSS in TM polarization: (**a**) $r_{a}$, (**b**) $r_{b}$, (**c**) $w_{a}$, and (**d**) $w_{b}$.

The effects of the design parameters on the stop and pass band characteristics are investigated by a parametric analysis. Figure 4 and Figure 5 present the transmission coefficient variation in the proposed R-FSS unit cell, alongside their optimum results, for the key design parameters: (a) $r_{a}\;(outer\;radius)$, (b) $r_{b}\;(inner\;radius)$, (c) $w_{a}$ (outer circle width) and (d) $w_{b}$ (inner circle width) for TE and TM modes, respectively. Figure 4a and Figure 5a analyze the effect of varying $r_{a}$ on $S_{21}$ under TE and TM polarizations, respectively, with results shown for Modes 1, 2, and 3. As $r_{a}$ increases, the associated inductance rises, resulting in a downward shift in the resonant frequency. In dual-band responses, this trend is more prominent in the first resonance, where the frequency decreases consistently with increasing $r_{a}$. Similarly, Figure 4b and Figure 5b analyze the effect of varying $r_{b}$ on the transmission coefficient for TE and TM polarizations, respectively. Since $r_{b}$ corresponds to the inner ring radius, its variation predominantly affects the second resonance in dual-band responses. As illustrated in Figure 4c and Figure 5c, an increase in $w_{a}$ leads to a shift in the resonant frequency toward higher values. Similarly, increasing the width of the inner ring in resonators leads to an upward shift in the resonant frequency, as shown in Figure 4d and Figure 5d.

**Figure 6:**
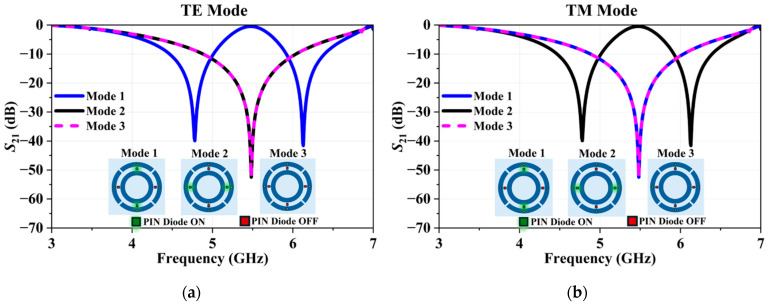
Transmission coefficient variation in the R-FSS for Modes 1, 2, and 3: (**a**) TE Mode and (**b**) TM Mode.

The overall design incorporates a unit cell with an area of 45 × 45 mm^2^. The metallic pattern, which consists of interconnected concentric rings of conductors, linked by polarization-aligned diodes, is designed on a RT/Duroid 5880 substrate. This substrate features a thickness of 1.575 mm, a relative permittivity of 2.2, and a loss tangent of 0.0019, signifying its desirable electrical properties. The conductive material is copper, with a thickness of 35 µm. All specific design parameters and dimensions of the unit cell are comprehensively detailed in Table 2. The PIN-diodes integrated into this design are BAP51-02 components manufactured by NXP. CST Microwave Studio is used for the full-wave analysis of the structure. For simulation purposes, the PIN-diodes are modeled using a lumped-component approach: series inductance and capacitance in the OFF mode, and series inductance and resistance in the ON mode. This model is constructed by strictly following the technical specifications and characteristic data provided in the BAP51-02 datasheet. This approach ensured that the simulation precisely reflected the component’s real-world behavior. The width of the pad to which the PIN-diode is connected has no significant effect on the S-parameters, as confirmed by parametric analysis, and is selected as 0.85 mm to facilitate soldering. The optimized R-FSS design parameters are given in Table 2. Simulated $S_{21}$ parameters of the R-FSS for Modes 1, 2, and 3 are illustrated in Figure 6.

Under TE polarization, Mode 1 exhibits two distinct stopbands: the first band 4.47–4.99 GHz with a resonance frequency of $f_{r1}=4.77$ GHz and a fractional bandwidth of 10.89%, and the second band 5.92–6.39 GHz with $f_{r2}=6.12$ GHz and a fractional bandwidth of 7.66%. Modes 2 and 3 under TE polarization both exhibit a single wideband characteristic in the range 4.90–6.05 GHz, corresponding to $f_{r}=5.50$ GHz and a fractional bandwidth of 20.91%. For TM polarization, Modes 1 and 3 also present single wideband from 4.90 to 6.05 GHz ($f_{r}=5.51$ GHz, 20.91% fractional bandwidth), whereas Mode 2 exhibits two stopbands identical to those of Mode 1 under TE polarization, with ranges of 4.47–4.99 GHz ($f_{r1}=4.77$ GHz, 10.89%) and 5.92–6.39 GHz ($f_{r2}=6.12$ GHz, 7.66%). These results demonstrate that the proposed reconfigurable FSS can selectively control the stopband distribution for different modes under TE and TM polarizations, thereby enabling polarization-dependent frequency response and maintaining stable performance across polarization states. The proposed structure has the ability to dynamically switch between two narrow stopbands and a single-wide stopband, as evidenced by the fractional bandwidth values obtained for each operating mode.

## 3. Equivalent Circuit Model (ECM) of the R-FSS

**Figure 7:**
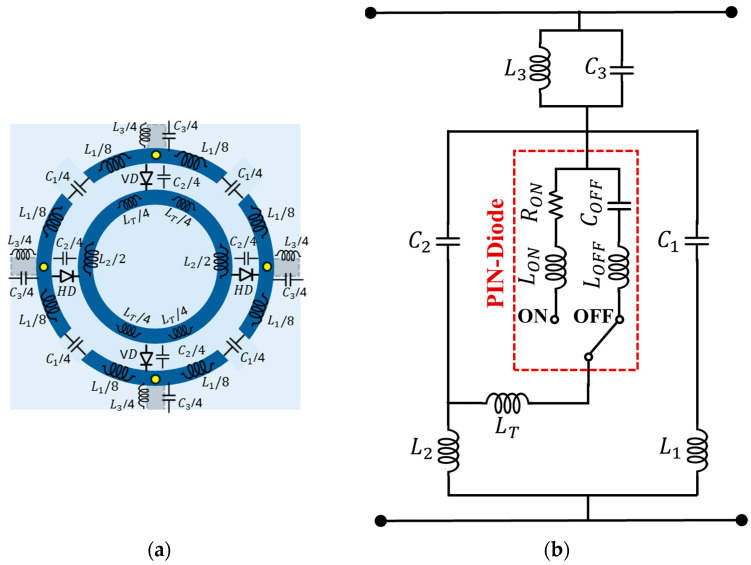
ECM of the proposed R-FSS: (**a**) lumped elements of the proposed R-FSS unit cell and (**b**) finalized ECM of the R-SS. The optimized values of inductors and capacitors are as follows: $L_{1}=\;9.147\;nH$, $L_{2}=\;15.77\;nH$, $C_{1}=\;0.225\;pF$, $C_{2}=\;0.0937\;pF$, $C_{3}=\;0.496\;pF$, $L_{3}=\;3.93\;nH$, $L_{T}=\;15.77\;nH$, $L_{ON}=\;0.75\;nH$, $C_{OFF}=\;0.2\;pF$, and $R_{ON}=\;1.5\;\Omega$.

An equivalent circuit model (ECM) analysis is carried out to validate the simulation results. The proposed R-FSS unit cell with the corresponding L–C elements are illustrated in Figure 7a, and its equivalent circuit model (ECM) is presented in Figure 7b.

**Figure 8:**
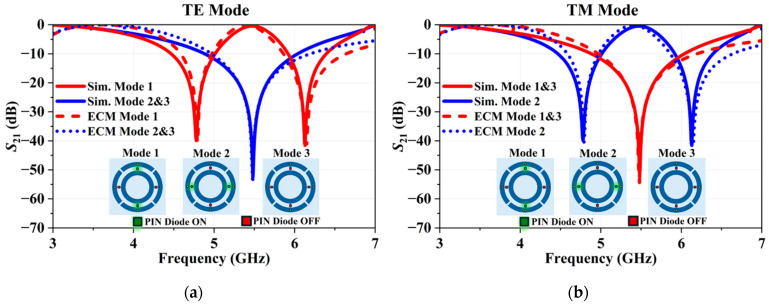
Comparison of the $S_{21}$ parameters: (**a**) TE polarization and (**b**) TM polarization.

Regarding the passive elements of the structure, the outer ring is modeled as an inductance ($L_{1}$), with the gaps on the outer ring represented by a series capacitance ($C_{1}$), while the gap and bottom-layer conductor (highlighted by gray rectangles) between the outer rings are modeled as a parallel inductance ($L_{3}$) and capacitance ($C_{3}$). Additionally, the inner ring is modeled as inductance ($L_{2}$ and $L_{T}$), while the gap between the rings is represented by a capacitance ($C_{2}$) in parallel with the PIN-diode. The accuracy of the ECM is validated by comparing the $S_{21}$ parameters of the simulated R-FSS and the simplified ECM, as shown in Figure 8. The small discrepancy outside the resonance region between the simulated and ECM curves may arise from the limitations of the equivalent circuit method in accurately capturing the complex electromagnetic interactions present in the frequency-selective surface.

## 4. Fabrication and Experimental Verification

**Figure 9:**
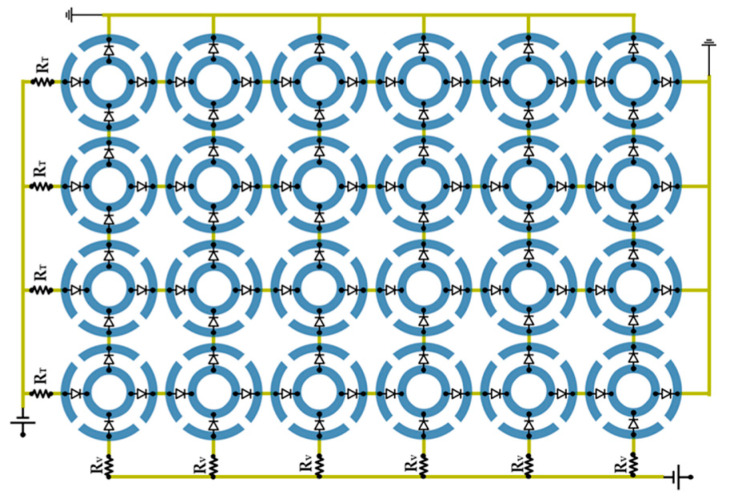
Schematic representation of the biasing diagram of the R-FSS.

To experimentally validate the simulation results, a prototype with dimensions of 208.6 × 284.8 mm^2^ and substrate thickness of 1.575 mm was fabricated utilizing printed circuit board (PCB) technology on Rogers 5880 dielectric substrate. The prototype consists of 4 × 6 array of unit cells. For ON/OFF control of the PIN-diodes placed in either horizontal or vertical orientations, the DC bias required is supplied from the bottom layer to the top layer through vias. The prototype integrates BAP-5102 PIN-diodes, with 8 vertically oriented and 12 horizontally oriented diodes soldered at their designated locations. Each diode requires a forward voltage of approximately 0.7 V ($V_{D})$ for forward biasing (ON state). The forward resistance of each PIN-diode is 1.5 Ω at a bias current of 10 mA. The current is limited to 10 mA ($I_{L})$ by surface-mount resistors (SMDs), designated as $R_{T}$ for horizontal diodes and $R_{V}$ for vertical diodes, as illustrated in Figure 9. To bias the vertically oriented diodes with a DC voltage of 9 V $\left(V_{DC}\right)$, a resistor value of $R_{V}$ = 340 Ω is required, whereas for the horizontally oriented diodes, $R_{T}$ = 60 Ω is expected, where the calculation of $R_{T}$ and $R_{V}$ values is presented in Equation (1). Consequently, the nearest commercial resistor values are chosen: 68 Ω for $R_{T}$ and 330 Ω for $R_{V}$.

(1) $$\frac{V_{DC}-\left(V_{D}*T_{N}\right)}{I_{L}}=R_{T}\;\&\;R_{V}$$

Here, $T_{N}$ denotes the total number of diodes, which is equal to 8 for vertically oriented diodes and 12 for horizontally oriented diodes. In Mode 1, only the vertical diodes are ON (9 V is applied to the vertical diodes). Mode 2 is achieved by turning the horizontal diodes ON (9 V is applied to the horizontal diodes). In Mode 3, all diodes are OFF. The schematic representation of the biasing diagram of the R-FSS is given in Figure 9.

**Figure 10:**
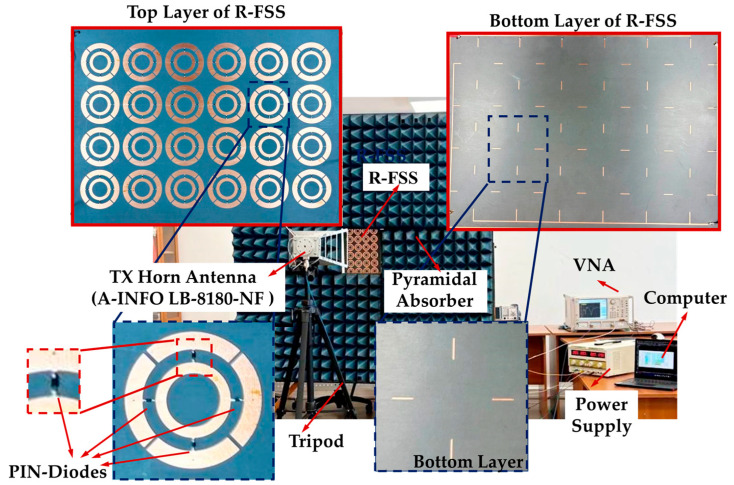
The measurement setup.

**Figure 11:**
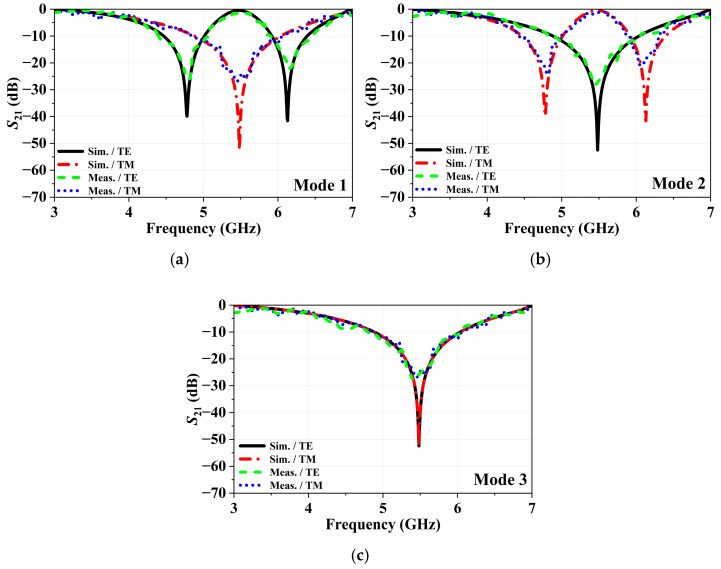
Measurement results of the R-FSS: (**a**) Mode 1, (**b**) Mode 2, and (**c**) Mode 3.

The measurement setup is given in Figure 10. For measurements, Anritsu MS4644A Vector Network Analyzer (VNA), capable of operating between 10 MHz and 40.5 GHz, is utilized. A TT Technic MCH303D-II DC power supply, with an adjustable output voltage range of 0–30 V, is employed to provide the required biasing voltage. The fabricated prototype is mounted in a cavity on a platform covered with pyramid absorbers, positioned between a pair of A-INFO LB-8180-NF horn antennas. To perform TM mode measurements, the sample is rotated 90° on the platform to ensure alignment with the desired polarization. Measurement results of R-FSS are given on Figure 11. The proposed structure exhibits measured band-stop characteristics with fractional bandwidths of 11.36% in the 4.43–4.97 GHz band and 6.78% in the 5.91–6.32 GHz band (Mode 1), and ~22.44% in the 4.82–6.03 GHz band (Mode 2 and 3) under TE polarization. For TM polarization, band-stop fractional bandwidths are 13.71% in the 4.41–5 GHz band and 8.95% in the 5.91–6.46 GHz band (Mode 2), and ~22.44% in the 4.85–6.07 GHz band (Mode 1 and 3).

The performance of an FSS is sensitive to the angle of incidence of the incoming electromagnetic wave. Consequently, it is essential to assess the angular stability of the FSS under oblique incidence conditions. The angular stability of frequency-selective surfaces is closely related to the electrical dimensions of the unit cell, as stated in Equation (2). It is well known that unit cells with reduced physical dimensions exhibit better performance in terms of angular stability. To ensure stability up to an incidence angle of $\theta_{0}$, the unit cell width, D, must satisfy the following criterion:

(2) $$D<\;\frac{\lambda_{0}}{1+sin\theta_{0}}$$

where $\lambda_{0}$ is the wavelength and $\theta_{0}$ is the angle of incidence [30]. The proposed structure, which incorporates PIN-diodes between two closed circular loops, is designed to switch between dual-narrow stopbands and a single-wide stopband; however, as indicated in Equation (2), the design parameters impose a requirement for a relatively large unit-cell size. This necessity inherently restricts the range of acceptable angles of incidence. The angular stability performance of the proposed unit cell was evaluated based on its resonance modes. For Mode 1 under TE polarization, two distinct resonances are observed at $f_{r1}=4.77$ GHz and $f_{r2}=6.12$ GHz. Using Equation (2), the corresponding angular stability limits are calculated as ${\theta_{0}}_{fr1}=23.35{}^{\circ}$ and ${\theta_{0}}_{fr2}=5.02{}^{\circ}$, respectively. These results indicate that angular stability up to approximately 25° can be achieved at ${fr}_{1}$, whereas stability is significantly reduced at ${fr}_{2}$. For Modes 2 and 3, at the resonance frequency of $f_{r}=5.5$ GHz, the ${\theta_{0}}_{fr}$ is calculated as 12.24°  implying that the stability can be maintained up to approximately 10°. The analysis of the proposed structure indicates no significant performance difference between TE and TM polarizations for arbitrary polarization states, demonstrating that the structure performs similarly in both horizontal and vertical polarization states. As stated in Equation (2), angular stability increases as the unit cell period D; therefore, to improve angular stability at the operating resonances of the proposed design, miniaturization techniques [44,45] are recommended to reduce D while maintaining the center frequency and bandwidth. A reduction in D enabling improved angular stability even for larger incidence angles of $\theta_{0}$.

**Figure 12:**
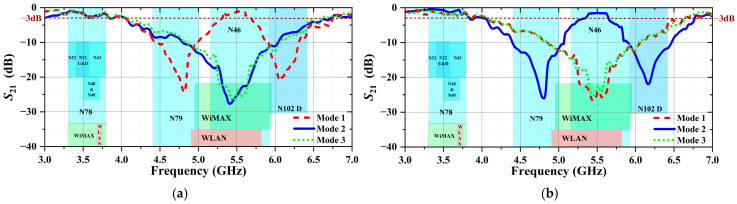
The corresponding frequency band definitions of the R-FSS based on measurement results: (**a**) TE Polarization and (**b**) TM Polarization.

The corresponding frequency ranges of the proposed R-FSS are summarized in Figure 12. In one mode, the structure suppresses two narrowbands corresponding to the n79/n102d allocations, whereas in the other mode, it suppresses a wideband covering the WiMAX, WLAN, and n46 allocations.

## 5. Conclusions

This study presents a novel reconfigurable frequency-selective surface (R-FSS) engineered for dynamic operation across multiple wireless communication standards, specifically WLAN, WiMAX, and sub-6 GHz frequency bands. Depending on the bias states of these diodes, the proposed structure transitions between three distinct operational modes. For TE polarization, Mode 1 has two narrowband-stop characteristics: one at 4.47–4.99 GHz with a 10.89% fractional bandwidth and another at 5.92–6.39 GHz with a 7.66% fractional bandwidth. Modes 2 and 3 exhibit a single wideband-stop characteristics at 4.90–6.05 GHz, with a 20.91% fractional bandwidth. Under TM polarization, Modes 1 and 3 also show this single-wide stopband characteristic, while Mode 2 displays the two stopband characteristics of Mode 1 under TE polarization. The fabricated prototype of the R-FSS demonstrates the effective operation of the structure in the WLAN, WiMAX, and sub-6 GHz frequency bands, confirming its functionality, as validated by ECM, numerical, and experimental results. The obtained results confirm the promising potential of this R-FSS structure, highlighting its adaptability for advanced wireless communication systems. To further enhance the angular stability and miniaturization of the proposed structure, future work could focus on an artificial neural network-driven optimization of geometric parameters for size reduction, aiming to refine its performance in various incidence angles.

## Figures and Tables

**Table 1:** Modes of the proposed R-FSS.

R-FSS Mode	Diode States	
	HD	VD
Mode 1	OFF	ON
Mode 2	ON	OFF
Mode 3	OFF	OFF

**Table 2:** Optimized R-FSS Design Parameters.

**Parameter**	W	L	$r_{a}$	$r_{b}$	$w_{a}$	$w_{b}$	$w_{c}$	$w_{g}$	$r_{via}$	$l_{c}$
**Value**	45	45	20.70	11.40	5.32	3.25	0.85	1	0.15	4.46

All units are in mm.

## Data Availability

The original contributions presented in this study are included in the article. Further inquiries can be directed to the corresponding author.

## Abbreviations

The following abbreviations are used in this manuscript:

R-FSS	Reconfigurable Frequency-Selective Surface
FSS	Frequency-Selective Surfaces
MEMS	Micro-Electromechanical Systems
